# The use of LeptiCore^® ^in reducing fat gain and managing weight loss in patients with metabolic syndrome

**DOI:** 10.1186/1476-511X-9-20

**Published:** 2010-02-19

**Authors:** Dieudonne Kuate, Blanche CO Etoundi, Boris KG Azantsa, Anne-Pascale N Kengne, Judith L Ngondi, Julius E Oben

**Affiliations:** 1 Laboratory of Nutrition & Nutritional Biochemistry, Department of Biochemistry, Faculty of Science, BP 8418, University of Yaounde 1, Yaounde, Cameroon

## Abstract

**Background:**

LeptiCore^® ^is a proprietary combination of various ingredients which have been shown to have properties which could be beneficial to weight loss in obese and overweight human subjects. This study evaluates the effect of Lepticore^® ^on bodyweight as well as parameters associated with obesity and metabolic syndrome.

**Methods:**

The study was an 8 week randomized, double-blind, placebo-controlled design involving 92 obese (mean BMI > 30 kg/m^2^) participants (37 males; 55 females; ages 19-52; mean age = 30.7). The participants were randomly divided into three groups: placebo (n = 30), LeptiCore^® ^formula A (low dose) (n = 31) and LeptiCore^® ^formula B (high dose) (n = 31). Capsules containing the placebo or active formulations were administered twice daily before meals with 300 ml of water. None of the participants followed any specific diet nor took any weight-reducing medications for the duration of the study. A total of 12 anthropomorphic and serological measurements were taken at the beginning of the study and after 2, 4, 6, and 8 weeks of treatment.

**Results:**

Compared to the placebo group, the two active groups showed statistically significant differences on all 12 variables by week 8. These included four anthropomorphic variables (body weight, body fat, waist and hip size) and eight measures of serological levels (plasma total cholesterol, LDL, HDL, triglycerides, blood glucose, serotonin, leptin, C-reactive protein). The two active groups also showed significant intra-group differences on all 12 variables between study onset and week 8.

**Conclusion:**

The LeptiCore^® ^formulation at both the low and high dosages appears to be helpful in the management of fat gain and its related complications. The higher dosage resulted in significantly greater reductions in body weight and triglyceride, blood glucose, and C-reactive protein levels, as well as increased serotonin levels.

## Background

Metabolic syndrome, a common disorder also known as Syndrome X and insulin resistance syndrome is (like many other obesity-related conditions) on the rise throughout the world. The American Heart Association criteria for metabolic syndrome include abdominal obesity, atherogenic dyslipidemia, elevated blood pressure, insulin resistance or glucose intolerance, prothrombotic state, and pro-inflammatory state.

As of 2001, some 47 million people in the United States had metabolic syndrome - which was projected to overtake cigarette smoking as the greatest risk factor of cardiovascular disease [1]. One of the accompanying conditions of metabolic syndrome that has not received as much attention is oxidative stress. This seems to be a key in the disease condition, and all efforts to address the independent components of metabolic syndrome should include an effort to reduce oxidative stress, which has not been clearly established to be a prelude or consequence of metabolic syndrome.

In an effort to delay the onset of heart disease and type 2 diabetes for the vast and ever-increasing numbers of overweight/obese persons now at high risk for these diseases, the current International Diabetes Foundation recommends pharmacological agents that specifically target individual components of metabolic syndrome [2-7]. Although these agents can have benefits, the combination of safety concerns, high costs, and the complex nature of the condition have encouraged the investigation of medicinal plants as natural supplements in its treatment.

LeptiCore^® ^is a proprietary blend of plant-based polysaccharides, esterified fatty acids, pomegranate, polyphenols and ellagic acid, beta carotene, and aphanizomenon flosaquae extract, which are components shown to reduce stored body fat, enhance weight loss, act as antioxidants and ameliorate the symptoms of metabolic syndrome.

Research has shown that leptin serves as a regulator of body fat storage by modulating satiation, glycemic control and metabolism; and that reduction in serum leptin correlates with lower regional body fat and total body fat [8-10]. Leptin has also been shown to cause oxidative stress [11], which can further exacerbate metabolic syndrome.

Leptin is secreted by fat - the more fat, the more leptin - yet it is named for the Greek word leptos, which means "thin." While obese people produce much higher levels of leptin than thin and normal-weight individuals, they are somehow resistant to its effects [8,12].

One reason for this may be that elevated levels of C-reactive protein (CRP), which inhibits leptin's role in controlling appetite [13]. CRP is now considered a key marker for low-grade systemic inflammation which, in turn, is considered by leading scientists to be responsible for a number of metabolic problems that result in unwanted weight gain and obesity [14]. Low grade, internal, invisible inflammation, oxidative stress, along with high leptin levels, may be a root cause of excess body fat and the inability to lose excess or unwanted fat [15].

*In vivo *and *in vitro *research done at the University of Pittsburgh demonstrated that CRP binds to leptin thereby impairing its signaling in two ways: 1) the coupling of CRP to leptin makes crossing the blood-brain barrier nearly impossible, thus preventing "free" leptin access to the hypothalamus; 2) the CRP/leptin complex inhibits the binding of leptin to its receptors, thus blocking its ability to signal in cultured cells. Since human CRP correlates with increased plasma leptin and adiposity, the results "suggest a potential mechanism to leptin resistance, by which circulating CRP binds to leptin and attenuates its physiological functions [16].

In one set of studies, the researchers delivered human leptin continuously for six days into mice which had receptors for leptin but lacked the ability to produce it. As expected, the plump mice ate less and lost weight, and their blood glucose levels normalized. Infusions containing both leptin and high doses of CRP, however, blocked the action of leptin. The plump mice just kept eating, getting fatter and fatter, and were no longer protected against diabetes. Giving CRP alone affected neither food intake nor body weight [16].

Another experiment showed that human liver cells increased CRP expression when exposed to leptin, which suggests that appetite may be regulated through a feedback loop that includes the liver in addition to the brain and leptin-secreting fat cells [16].

Of the various ingredients in the LeptiCore formulation, the plant-based polysaccharides and esterified fatty acids have been shown to lower leptin level, resulting in reduced appetite and weight loss [17,18]. Various studies have shown that pomegranate extract contains polyphenolic compounds and beta carotenes, which have considerable efficacy in lowering CRP, resulting in reduced inflammation and lowered cholesterol [19-21]. The reduction of inflammation and CRP promotes leptin's effectiveness, increasing the possibility of reduced appetite and weight loss. These components have also been shown to be effective antioxidants which will consequently improve the components of metabolic syndrome.

The last of the natural ingredients in this formulation, the phycocyanamines (blue green algae--a high phenylethylamine (PEA) blend) addresses the emotional aspects of metabolic syndrome by increasing the vital neurotransmitter, serotonin (5-HT), a well known mood elevator [22,23]. Many studies have shown that serotonin affects eating behavior and body weight, and that increased serotonin plasma levels are associated with decreased food intake, reduced weight gain, and increased energy expenditure [24,25].

The primary purpose of the present study was to determine the effects of two dosage levels of a unique, natural formulation containing the above agents on weight loss in patients with metabolic syndrome.

## Methods

### Participants

Ninety two obese participants were recruited for the 8-week study. Based on physical examination by a physician and laboratory screening tests, all participants met the American Heart Association criteria for metabolic syndrome, which include abdominal obesity, atherogenic dyslipidemia, elevated blood pressure, insulin resistance or glucose intolerance, prothrombotic state, and pro-inflammatory state.

Specific inclusion criteria included: BMI > 30 kg/m^2^; total cholesterol >200 mg/dl; LDL cholesterol >160 mg/dl; HDL cholesterol <40 mg/dl; triglycerides >150 mg/dl; fasting blood glucose >100 mg/dl; and blood pressure >130/85 mmHg.

Specific exclusion criteria included: morbid obesity (BMI > 40 kg/m^2^); diabetes mellitus requiring daily insulin management; pregnancy/lactation; active infection; and systemic disease such as HIV/AIDS, active hepatitis or clinical signs of active malignancy within the past 5 years. Other exclusion criteria included the use of any cholesterol-lowering medications 30 days prior to enrolling in the study and/or medication (e.g., steroids) that interfere with healing; and enrolment in another clinical study within the past 6 months.

Of the 92 subjects, 37 were male and 55 were female. The age range was 19-52 (mean age = 30.7), and the mean BMI was > 30 kg/m^2^.

The purpose, nature and potential risks of the study were explained to all patients and all gave a written informed consent before participation. The Cameroon National Ethics Committee approved the protocol. The study was conducted in accordance with the Helsinki Declaration (1983 version). None of the participants took any weight-reducing medication nor followed any specific diet during the duration of the study.

### Study design/Intervention

The study was a randomized, double-blind, placebo-controlled design. Participants were randomly divided into three groups: placebo, LeptiCore^® ^formula A (low dose) and LeptiCore^® ^formula B (high dose). The placebo or active formulations were administered twice daily before meals with approximately 300 ml (8-10 oz.) of water. Formula A = 1 capsule (300 mg each); Formula B = 2 capsules (300 mg each). Since the active and placebo capsules were identical in shape, color, and appearance, neither the participants nor the researchers knew which capsule was administered.

All product formulations were supplied by Pipeline Nutraceuticals, Inc. Fairfield, California, USA. All encapsulating, bottling and packaging was supplied by and carried out by Protein Research, Inc., Livermore, California, USA.

During the study period, subjects were examined bi-weekly. Their body weight, percent body fat, and waist/hip circumferences were recorded, and serological analysis was performed. Subjective impressions (e.g., increased/decreased appetite, feelings of lightness, gastrointestinal pains, etc.) were solicited and recorded during each visit. The subjects were also asked about their physical activity and food intake although no major dietary changes or exercises were suggested.

#### Anthropometric measurements

Body weight, body fat, waist and hip circumference were assessed at each visit with a Tanita™ BC-418 Segmental Body Composition Analyzer/Scale that uses bio-electrical impedance analysis for body composition analysis. Height was measured with a Harpended™ stadiometer, which measures the length of curved line staffage to the nearest 0.5 cm.

Participants (12-hour fasted) were encouraged to wear light clothing before measurements were taken. Waist and hip measures were obtained with a soft non-stretchable plastic tape. Waist circumference was measured on the narrowest and the widest parts of the trunk; hip circumference was obtained at the widest point of the hip. In an effort to ensure intra-individual consistency, the participants were measured at approximately the same time of day each visit.

#### Serological/Laboratory methods

Blood samples were collected after a 12-hour overnight fast into heparinized tubes at the beginning of the study and after 2, 4, 6, and 8 weeks of treatment. The concentrations of total cholesterol, LDL cholesterol, HDL cholesterol, triglycerides, fasting blood glucose, serotonin, C-reactive protein, and leptin were measured using commercial diagnostic kits from SIGMA Diagnostics, St. Louis, Missouri, USA.

#### Statistical Analysis

The data for each parameter were summarized (n, mean, standard deviation) for Week 0 (Initial) and Week 8 (final) and for the intra-group percent differences. The percent change from baseline was tested for differences using the Mixed Effects Model, a flexible tool for analyzing repeated and longitudinal treatments.

## Results

### Anthropomorphic characteristics (body weight, body fat, waist size, hip size)

As shown in Tables 1, 2, 3, and 4, over a period of 8 weeks the two treatment groups (Formula A = low dose; Formula B = high dose) showed a statistically significant decrease compared to the placebo group on all four variables. The only significant difference between the two formulations was in body weight loss (see Table 1).

### Body weight (Table 1)

Although the placebo group showed virtually no change (0.82 kg) in body weight over the 8-week trial, the Formula A group lost 5.2 kg (p < 0.001), and the Formula B group lost 6.6 kg (p < 0.001). In terms of intra-group mean-% change from baseline (Initial) to 8 weeks, the placebo, Formula A and Formula B groups lost 0.82%, 5.4% (p < .05), and 6.95% (p < 0.001), respectively.

**Table 1:** Body weight: effectiveness of treatments

	Body Weight (mean kg)		Weight change (%)
	**Initial**	**8 Weeks**	**8 Weeks - Initial**

**Formula B (↑dose)**	100.93 ± 6.30	94.36 ± 5.67^b^*	-6.95^‡^

**Formula A (↓dose)**	102.28 ± 7.17	97.04 ± 5.95^b^	-5.40^†^

**Placebo**	101.32 ± 6.13	100.50 ± 7.28	-0.82

^a^p < 0.05; ^b^p < 0.001 compared with **Placebo**

*p < 0.05; **p < 0.001 compared with **Formula A**

^†^p < 0.05; ^‡^p < 0.001 compared with **Initial**; intragroup analysis

### Body fat (Table 2)

As with body weight, the placebo group showed little (0.1%) change in mean %-body fat after 8 weeks whereas the Formula A group lost 1.1% (p < 0.05), and the Formula B group lost 1.8% (p < 0.05). In terms of intra-group mean % change from baseline to 8 weeks, the placebo, Formula A and Formula B groups lost 0.24%, 2.8% (p < 0.05), and 4.1% (p < 0.001), respectively.

**Table 2:** Body fat: effectiveness of treatments

	Body fat (mean %)		Fat reduction (%)
	**Initial**	**8 Weeks**	**8 Weeks - Initial**

**Formula B (↑dose)**	43.85 ± 5.60	42.04 ± 6.63^a^	-4.13^‡^

**Formula A (↓dose)**	40.22 ± 7.26	39.08 ± 6.12^a^	-2.83^†^

**Placebo**	42.28 ± 8.27	42.18 ± 8.117	-0.24

^a^p < 0.05; ^b^p < 0.001 compared with **Placebo**

*p < 0.05; **p < 0.001 compared with **Formula A**

^†^p < 0.05; ^‡^p < 0.001 compared with **Initial**; intragroup analysis

### Waist size (Table 3)

Waist circumference is one of the most important determinants in the diagnosis of obesity and metabolic syndrome. In this case, the Formula A and Formula B groups showed a 4.6 cm and 5.6 cm decrease, respectively, vs. the 0.6 cm decrease demonstrated by the placebo group (p < 0.05). In terms of intra-group mean % change in waist size from baseline to 8 weeks, the placebo, Formula A and Formula B groups lost 0.57%, 4.4% (p < 0.05), and 4.6% (p < 0.001), respectively.

**Table 3:** Waist size: effectiveness of treatments

	Waist circumference (mean cm)		Waist change (%)
	**Initial**	**8 Weeks**	**8 Weeks - Initial**

**Formula B (↑dose)**	110.50 ± 7.28	105.38 ± 10.64^a^	-4.64^‡^

**Formula A (↓dose)**	104.00 ± 8.63	99.40 ± 11.73^a^	-4.42^†^

**Placebo**	105.60 ± 7.91	105.00 ± 15.77	-0.57

^a^p < 0.05; ^b^p < 0.001 compared with **Placebo**

*p < 0.05; **p < 0.001 compared with **Formula A**

^†^p < 0.05; ^‡^p < 0.001 compared with **Initial**; intragroup analysis

### Hip size (Table 4)

Once again, although the placebo group showed virtually no change (0.4 cm), the Formula A group lost 4.4 cm (p < 0.05), and the Formula B group lost 5.6 cm (p < 0.05) in circumference over the 8-week trial. In terms of intra-group mean % change from baseline to 8 weeks, the placebo, Formula A and Formula B groups lost 0.32%, 3.5% (p < .05), and 4.6% (p < 0.001), respectively.

**Table 4:** Hip size: effectiveness of treatments

	Hip circumference (mean cm)		Hip change (%)
	**Initial**	**8 Weeks**	**8 Weeks - Initial**

**Formula B (↑dose)**	123.38 ± 10.84	117.75 ± 17.24^a^	-4.56^‡^

**Formula A (↓dose)**	126.20 ± 12.91	121.80 ± 15.41^a^	-3.49^†^

**Placebo**	125.85 ± 14.72	125.45 ± 17.99	-0.32

^a^p < 0.05; ^b^p < 0.001 compared with **Placebo**

*p < 0.05; **p < 0.001 compared with **Formula A**

^†^p < 0.05; ^‡^p < 0.001 compared with **Initial**; intragroup analysis

### Serological characteristics (total cholesterol, LDL cholesterol, HDL cholesterol, triglycerides, fasting blood glucose, serotonin, leptin, C-reactive protein)

As shown in Tables 5, 6, 7, 8, 9 and 10, the two treatments (vs. placebo) groups showed a statistically significant difference on all eight variables by the end of the 8-week trial period. There were significant differences between the two formulations (in favour of the high dosage Formula B) on triglyceride, blood glucose, serotonin, and C-reactive protein levels.

### Plasma total cholesterol level (Table 5)

In contrast to the small (4.5 mg/dL) reduction in total cholesterol shown by the placebo group at 8 weeks, the reduction for the Formula A group was 37.6 mg/dL (p < 0.05), and that of the Formula B group was 57.9 mg/dL (p < 0.001). In terms of intra-group mean % change from baseline (Initial) to 8 weeks, the placebo group decrease was 2.1% whereas the Formula A and Formula B groups decreases were 18.0% (p < 0.05) and 27.5 (p < 0.001), respectively.

**Table 5:** Plasma Total Cholesterol, LDL cholesterol and HDL cholesterol levels: effectiveness of treatments

		Time (weeks)		% Change
**Variable**	**Group**	**Initial**	**8**	**8 weeks - Initial**
Total Cholesterol (mg/dl)	Placebo	211.16 ± 11.92	206.68 ± 20.19	-2.12
	Formula A (**↓**dose)	208.74 ± 10.49	171.17 ± 18.82^a^	-18.00^†^
	Formula B (**↑**dose)	210.64 ± 9.30	152.75 ± 10.19^b^	-27.48^‡^
				
LDL Cholesterol (mg/dl)	Placebo	166.21 ± 9.25	162.63 ± 17.83	-2.39
	Formula A (**↓**dose)	163.94 ± 8.27	139.91 ± 8.92^a^	-14.66^†^
	Formula B (**↑**dose)	165.84 ± 7.29	133.05 ± 7.71^b^	-19.77^‡^
				
HDL Cholesterol (mg/dl)	Placebo	35.92 ± 5.16	37.22 ± 5.59	+3.62
	Formula A (**↓ **dose)	34.72 ± 3.28	39.46 ± 4.48^a^	+13.65^†^
	Formula B (**↑**dose)	32.43 ± 2.10	38.63 ± 3.11^b^	+19.12^‡^

^a^p < 0.05; ^b^p < 0.001 compared with **Placebo**

*p < 0.05; **p < 0.001 compared with **Formula A**

^†^p < 0.05; ^‡^p < 0.001 compared with **Initial**; intragroup analysis

### Plasma LDL cholesterol level (Table 5)

As with total cholesterol level, the placebo group showed a small (3.6 mg/dL) decrease in LDL cholesterol by 8 weeks. This can be compared with the 24.0 mg/dL reduction shown by the Formula A group (p < 0.05) and the 32.8 mg/dL reduction of the Formula B group (p < 0.001). In terms of intra-group mean % change in LDL from baseline to 8 weeks, the placebo, Formula A and Formula B groups decreased by 2.4%, 14.7% (p < 0.05), and 19.8% (p < 0.001), respectively.

### Plasma HDL cholesterol level (Table 5)

On one of the few variables where an increase in level is desirable, the placebo group showed a very small (1.3 mg/dL) increase in HDL cholesterol at 8 weeks. This can be compared with the 4.7 mg/dL increase shown by the Formula A group (p < 0.05) and the 6.2 mg/dL increase of the Formula B group (p < 0.001). In terms of intra-group mean % change in HDL from baseline to 8 weeks, the placebo, Formula A and Formula B groups increased by 3.6%, 13.6% (p < 0.05), and 19.1% (p < 0.001), respectively.

### Plasma triglyceride level (Table 6)

Per the above, the placebo group demonstrated a small change (1.0 mg/dL decrease) in triglyceride level at the end of the 8-week trial. This can be compared with the 11.5 mg/dL decrease shown by the Formula A group (p < 0.05) and the 27.6 mg/dL reduction demonstrated by the Formula B group (p < 0.001). In terms of intra-group mean % change in triglyceride level from baseline to 8 weeks, the placebo, Formula A and Formula B groups decreased by 0.6%, 7.1% (p < 0.05), and 16.4% (p < 0.001), respectively.

**Table 6:** Plasma Triglyceride level: effectiveness of treatments

	Triglyceride (mean mg/dl)		change (%)
	**Initial**	**8 Weeks**	**8 Weeks - Initial**

**Formula B (↑dose)**	167.61 ± 10.84	140.04 ± 4.29^b^*	-16.45^‡^

**Formula A (↓dose)**	162.21 ± 12.95	150.71 ± 8.30^a^	-7.09^†^

**Placebo**	168.93 ± 14.04	167.90 ± 10.30	-0.61

^a^p < 0.05; ^b^p < 0.001; compared with **Placebo**

*p < 0.05; **p < 0.001; compared with **Formula A**

^†^p < 0.05; ^‡^p < 0.001 compared with **Initial**; intragroup analysis

### Fasting blood glucose levels (Table 7)

In contrast to the small (1.6 mg/dL) decrease in blood glucose level shown by the placebo group at 8 weeks, the reduction for the Formula A group was 8.4 mg/dL (p < 0.05) and that of the Formula B group was 12.8 mg/dL (p < 0.001). In terms of intra-group mean % change from baseline to 8 weeks, the placebo, Formula A and Formula B groups decreased by 1.5%, 7.9% (p < 0.05), and 12.9% (p < 0.001), respectively.

**Table 7:** Fasting Blood Glucose levels: effectiveness of treatments

	Blood Glucose (mean mg/dl)		change (%)
	**Initial**	**8 Weeks**	**8 Weeks - Initial**

**Formula B (↑dose)**	104.84 ± 9.39	92.06 ± 6.29^b^*	-12.19^‡^

**Formula A (↓dose)**	105.61 ± 8.62	97.22 ± 7.81^a^	-7.94^†^

**Placebo**	107.52 ± 8.73	105.90 ± 10.79	-1.51

^a^p < 0.05; ^b^p < 0.001; compared with **Placebo**

*p < 0.05; **p < 0.001 compared with **Formula A**

^†^p < 0.05; ^‡^p < 0.001 compared with **Initial**; intragroup analysis

### Serum serotonin levels (Table 8)

In another case (see HDL cholesterol above) where an increase in level is desirable, the placebo group showed a very small (1.6 mg/dL) increase in serotonin levels at 8 weeks. This can be compared with the 9.9 mg/dL increase shown by the Formula A group (p < 0.05) and the 13.7 mg/dL increase of the Formula B group (p < 0.001). In terms of intra-group mean % change in serotonin from baseline to 8 weeks, the placebo, Formula A and Formula B groups increased by 9.1%, 28.6% (p < 0.001), and 38.6% (p < 0.001), respectively.

**Table 8:** Serum Serotonin levels: effectiveness of treatments

	Serotonin (mean mg/dl)		change (%)
	**Initial**	**8 Weeks**	**8 Weeks - Initial**

**Formula B (↑dose)**	35.40 ± 2.62	49.06 ± 2.85^b^*	38.59^‡^

**Formula A (↓dose)**	34.55 ± 2.85	44.42 ± 2.96^a^	28.57^‡^

**Placebo**	34.20 ± 3.15	37.31 ± 3.69	9.09

^a^p < 0.05; ^b^p < 0.001 compared with **Placebo**

*p < 0.05; **p < 0.001 compared with **Formula A**

^†^p < 0.05; ^‡^p < 0.001 compared with **Initial**; intragroup analysis

### Serum leptin levels (Table 9)

In contrast to the 4.4 mg/dL decrease in serum leptin levels shown by the placebo group at 8 weeks, the reduction for the Formula A group was 14.8 mg/dL (p < 0.05) and that of the Formula B group was 14.38 mg/dL (p < 0.001). In terms of intra-group mean % change from baseline to 8 weeks, the placebo, Formula A and Formula B groups decreased by 14.4%, 47.0% (p < 0.001), and 48.0% (p < 0.001), respectively.

**Table 9:** Serum Leptin levels: effectiveness of treatments

	Leptin (mean mg/dl)		change (%)
	**Initial**	**8 Weeks**	**8 Weeks - Initial**

**Formula B (↑dose)**	30.15 ± 1.50	15.83 ± 1.13^b^	-47.59^‡^

**Formula A (↓dose)**	31.52 ± 1.21	16.72 ± 1.40^a^	-46.95^‡^

**Placebo**	30.33 ± 1.63	25.97 ± 1.61	-14.38

^a^p < 0.05; ^b^p < 0.001 compared with **Placebo**

*p < 0.05; **p < 0.001; compared with **Formula A**

^†^p < 0.05; ^‡^p < 0.001 compared with **Initial**; intragroup analysis

### C-reactive protein levels (Table 10)

Per the above, the placebo group demonstrated a small change (0.4 mg/l decrease) in C-reactive protein levels at the end of the 8-week trial. This can be compared with the 1.6 mg/l decrease shown by the Formula A group (p < 0.05) and the 3.4 mg/l reduction demonstrated by the Formula B group (p < 0.001). In terms of intra-group mean % change in blood levels of C-reactive protein from baseline to 8 weeks, the placebo, Formula A and Formula B groups decreased by 3.3%, 15.1% (p < 0.001), and 29.4% (p < 0.001), respectively.

**Table 10:** C-Reactive Protein levels: effectiveness of treatments

	CRP (mean mg/l)		change (%)
	**Initial**	**8 Weeks**	**8 Weeks - Initial**

**Formula B (↑dose)**	11.58 ± 1.11	8.17 ± 0.61^b^*	-29.45^‡^

**Formula A (↓dose)**	10.99 ± 1.26	9.33 ± 0.43^a^	-15.10^‡^

**Placebo**	12.46 ± 1.30	12.05 ± 1.39	-3.31

^a^p < 0.05; ^b^p < 0.001 compared with **Placebo**

*p < 0.05; **p < 0.001 compared with **Formula A**

^†^p < 0.05; ^‡^p < 0.001 compared with **Initial**; intragroup analysis

### Adverse events

Adverse events with an incidence >3 included lack of sleep (4), and headache (4). Since the type and incidence of all reported side effects were also observed in the placebo group, one can assume that the LeptiCore^® ^formulation had few, if any, negative side effects.

## Discussion

Our results support the hypothesis that the use of LeptiCore^®^, a unique natural formulation, has considerable efficacy on weight loss in patients with metabolic syndrome. Compared to the placebo group, the two active groups (low and high dosage) showed statistically significant differences on all 12 variables by the end of the 8-week trial. Although there were no differences between the two active groups on body fat, waist and hip size; or cholesterol (total, HDL, LDL) or leptin levels, the higher dosage did result in significantly greater reductions in body weight, and triglyceride, blood glucose, CRP and serotonin levels.

It appears that LeptiCore^® ^somehow enhances cell membrane stability for improved cellular communication between the liver and adipose tissues for enhanced fatty acid utilization leading to regional fat loss. In addition, modulation of membrane-based inflammatory markers helped to reduce the inflammatory component of fat gain. This is where this formulation differs from other leptin-reducing supplements. Ingredients in this proprietary blend not only lowered serum leptin levels but also reduced CRP, a key marker for low grade inflammation. As discussed earlier, some researchers believe the binding of CRP to leptin may be the reason [16]. The fact that CRP is elevated in obese people increases the plausibility of their argument. CRP, which is produced by the liver and typically rises as a part of the immune system's inflammatory response, has been gaining favor as a marker for hypertension and heart disease risk-- both known complications of obesity.

LeptiCore's efficacy may also be due to the fact that it effects a time-dependent transit through the gastro-intestinal tract which, in turn, elicits a signal to regulate leptin levels. This is the mechanism for reducing the level of leptin in the bloodstream and the corresponding reduction in the percentage of body fat. LeptiCore's ability to lower CRP levels allows the remaining circulating leptin to reside in its "free" state and bind to appetite-regulating leptin receptors in the hypothalamus.

Yet another aspect of LeptiCore's action is its potential antioxidant power. The plasma concentrations of leptin are markedly increased in human obesity and positively correlated to body fat mass [26]. As human obesity is associated with hyperleptinemia and atherosclerosis, it was shown that leptin, in addition to its angiogenic properties, exerts proatherogenic effect on endothelial cells by increasing reactive oxygen species (ROS) formation. As mentioned earlier, Boulimie et al. [11] have shown that the administration of leptin may stimulate increases in oxidative stress *in vitro *cultured human endothelial cells. Increases in oxidative stress in the vascular endothelium may interact with nitric oxide to form peroxynitrite and thereby decrease the bioavailability of nitric oxide, which, in turn, could reduce weight gain and food intake [27].

## Competing interests

The authors declare that they have no competing interests.

## Authors' contributions

JEO conceived, co-designed and coordinated the study as well as prepared the manuscript; JLN co-designed and worked on the initial draft of the manuscript; DK carried out anthropometric measurements, analytical work and statistical analysis, BCOE carried out analytical work; BGKA carried out analytical work; ANK carried out analytical and statistical analysis. All authors read and approved the final manuscript.
